# Impact of a nanofiltration system on microplastic contamination in Geneva groundwater (Switzerland)

**DOI:** 10.1007/s11356-024-31940-y

**Published:** 2024-01-23

**Authors:** Angel Negrete Velasco, Alicia Ellero, Stéphan Ramseier Gentile, Stéphane Zimmermann, Pascal Ramaciotti, Serge Stoll

**Affiliations:** 1 Department F.-A. Forel for Environmental and Aquatic Sciences, Faculty of Science, Group of Environmental Physical Chemistry, University of Geneva, 66, Boulevard Carl-Vogt, CH-1211 Geneva 4, Switzerland; 2 SIG, Industrial Boards of Geneva, Geneva, Switzerland

**Keywords:** Microplastics, Fibres, Groundwater, Nanofiltration, Infrared microscopy, Suspended particulate matter

## Abstract

Microplastics (MPs) have been observed in the oceans, fresh waters, karstic water and remote water bodies. However, little is known on groundwater contamination, which is a natural resource of utmost importance for millions of people and is often perceived as a reliable source of water. Moreover, nanofiltration is perceived as a reliable technology to remove contaminants from water. In this study, large sample volumes of a silty-sandy gravel aquifer and the corresponding nanofiltered water were analysed for the presence of MPs (> 20 µm) using Fourier transform infrared (FTIR) microscopy. Concentration in ground water was 8 ± 7 MPs/m^3^ and increased to 36 ± 11 MPs/m^3^ in nanofiltered water. All MPs had a maximum Ferret diameter lower than 500 µm. Size distribution of MPs was towards the small size class (20–50 µm). In groundwater, 33% of MPs were detected in the smallest size class (20–50 µm) and 67% in the 50–100-µm-size class. In comparison, around 52% of MPs in nanofiltered water were observed in the 20–50 µm size class. Moreover, 33% of the MPs observed in nanofiltered water were in the 50–100 µm size class and 15% in the 100–500-µm-size class. From a chemical point of view, different plastic polymers were identified in groundwater and in nanofiltered water, such as polypropylene (PP), polyvinyl chloride (PVC), ethylene (vinyl acetate) copolymer (EVA), polyethylene (PE), polyethylene terephthalate (PET), polymethyl methacrylate (PMMA) and other polymer materials (such as polystyrene-based copolymers, vinyl-based copolymers). Fibres were observed in all samples, but only a small number of fibres (near 1%) were identified as PP synthetic fibres in nanofiltered water. Furthermore, no clear difference of fibre concentrations was observed between groundwater (232 ± 127 fibres/m^3^) and nanofiltered water (247 ± 118 fibres/m^3^). Groundwater had extremely low levels of microplastics, and although the nanofiltration effectively removes suspended particulate matter, it slightly contaminates the filtered water with MPs.

**Supplementary Information:**

The online version contains supplementary material available at 10.1007/s11356-024-31940-y.

## Introduction

Microplastics (MPs) have been defined as “any solid plastic particle insoluble in water with any dimension between 1 µm and 5 mm” (International Organisation for Standarization 2020). These MPs have been observed since the early 1970s (Carpenter et al. 1972; Colton et al. 1974) in the oceans and have been investigated in other aquatic environments such as fresh waters (Eerkes-Medrano et al. 2015; Mani et al. 2015; Shen et al. 2021), karstic water (Panno et al. 2019) and remote water bodies (Free et al. 2014; González-Pleiter et al. 2020; Negrete Velasco et al. 2020). MPs have also been reported in the air (Dris et al. 2017; Gasperi et al. 2018), in some food products (Liebezeit and Liebezeit 2014; Kosuth et al. 2018) and in drinking water (Chapa-Martínez et al. 2016; Mason et al. 2016; Carrington 2017; Dalmau-Soler et al. 2021). First, estimates point to a weekly intake between 0.1 and 5 g of MPs, with drinking water being the main source of MP ingestion (Senathirajah et al. 2021). A probabilistic lifetime exposure model for children and adults calculated that median MP intake for children is 553 particles/capita/day (184 ng/capita/day) and for adults the number increase to 883 particles/capita/day (583 ng/capita/day) (Mohamed Nor et al. 2021).

The presence of MPs in drinking water sources, at every stage of the drinking water treatment and distribution processes, as well as in tap water, is of great concern for the scientific community, the private and public sectors because of the potential implications for human health (WHO 2017, 2022; SAPEA 2019; Coffin and Weisberg 2022; Coffin et al. 2022). It has been demonstrated that human exposure to MPs results in acute and/or chronic lung inflammation (when inhaled) (Pauly et al. 1998), absorption of particles into the bloodstream (Leslie et al. 2022) and cytotoxicity at cell level (Schirinzi et al. 2017). Therefore, it is necessary to examine actual exposure, potential pathways, assess drinking water sources and MP removal efficiencies, especially in drinking water treatment processes. In this regard, conventional drinking water treatment (coagulation-flocculation, sedimentation, sand filtration and activated carbon filtration) work to reduce MPs from water (Novotna et al. 2019; Negrete Velasco et al. 2022), but membrane filtrations are promising technologies for MP removal, which deserves further attention (Shen et al. 2020).

Groundwater is a natural resource of utmost importance used worldwide for many activities and is often perceived as a reliable source of water. Groundwater requires indeed typically few or no treatment in comparison with surface waters for purification. However, groundwater is susceptible to contamination by agriculture, industry, sewage, contaminated water bodies’ infiltration and landfills’ infiltration (Carr et al. 2016; Dyachenko et al. 2017; Bharath et al. 2021) with heavy metals, salinity, coliform and emerging contaminants (such as microplastics) (UNESCO World Water Assessment Programme 2022). Indeed, high concentrations of MPs (between 3 and 23 MPs/L) were found in groundwater in Chennai (India) due to water infiltration near deposal sites (Bharath et al. 2021). In this regard, advanced water treatments, such as membrane filtration (microfiltration, ultrafiltration, nanofiltration or reverse osmosis), can be used to significantly reduce suspended particulate concentrations and emerging contaminants from water (including micropollutants from agricultural wastewater, polycyclic aromatic hydrocarbons and antibiotics) (Van der Bruggen et al. 2001; Li et al. 2019; Ren et al. 2022). These technologies have been shown to be efficient in removing MPs in highly contaminated waters and improving water quality such as wastewater treatment plant effluents (Ziajahromi et al. 2017; Akarsu et al. 2021), landfill leachates (Kara et al. 2022; Singh et al. 2023) and after conventional drinking water treatment of surface waters (Barbier et al. 2022). Nevertheless, little is known about the impact of nanofiltration units in low contaminated water, such as groundwater, with MPs. It is important to keep in mind that although the contamination levels of water with MPs are not yet regulated, the fact that there are MPs in drinking water sources and treated waters should not be neglected. Research addressing the impact of nanofiltration systems specifically in groundwater is very limited so far. The goal of this study is to evaluate MP contamination (> 20 µm) in groundwater from a silty-sandy gravel aquifer and the impact of a nanofiltration system (Station de Soral, Geneva, Switzerland) on MP contamination. To that end, existing guidelines on MP analysis (Schymanski et al. 2018; Koelmans et al. 2019; Brander et al. 2020; Cowger et al. 2020) were followed as much as possible to ensure reproducibility, comparability and quality. Three sampling campaigns of large water samples (500–1000 L) were analysed, contrary to several studies which investigated limited volumes or made single analysis.

## Materials and methods

### Sampling site

Industrial Boards of Geneva (*Services Industriels de Genève — SIG)* supplies near 55 million m^3^ drinking water per year in Geneva (Switzerland). Currently, around 10% of Geneva’s drinking water is extracted from groundwater bodies. Genevese aquifer (*Nappe du Genevois*) is the most important underground drinking water reserve in the canton of Geneva. This is a natural resource shared by the canton of Geneva, Switzerland, and the department of Haute-Savoie, in France. The aquifer is mainly fed by the waters of the Arve River and an artificial recharging station, which injects water drawn directly from the Arve into the underground aquifer. The groundwater is contained in an aquifer consisting of silty-sandy gravel of glacial and fluvioglacial origins (Wurm), lying directly on the molasse formation (impermeable substratum). This aquifer formation is overlaid by a clayish Wurmian moraine, which has the advantage of providing natural protection from surface contaminations. The Darcy permeability of the aquifer is around 1–2.10^−3^ m/s, but it could reach up to 3.10^−2^–5.10^−7^ m/s.

**Fig. 1:**
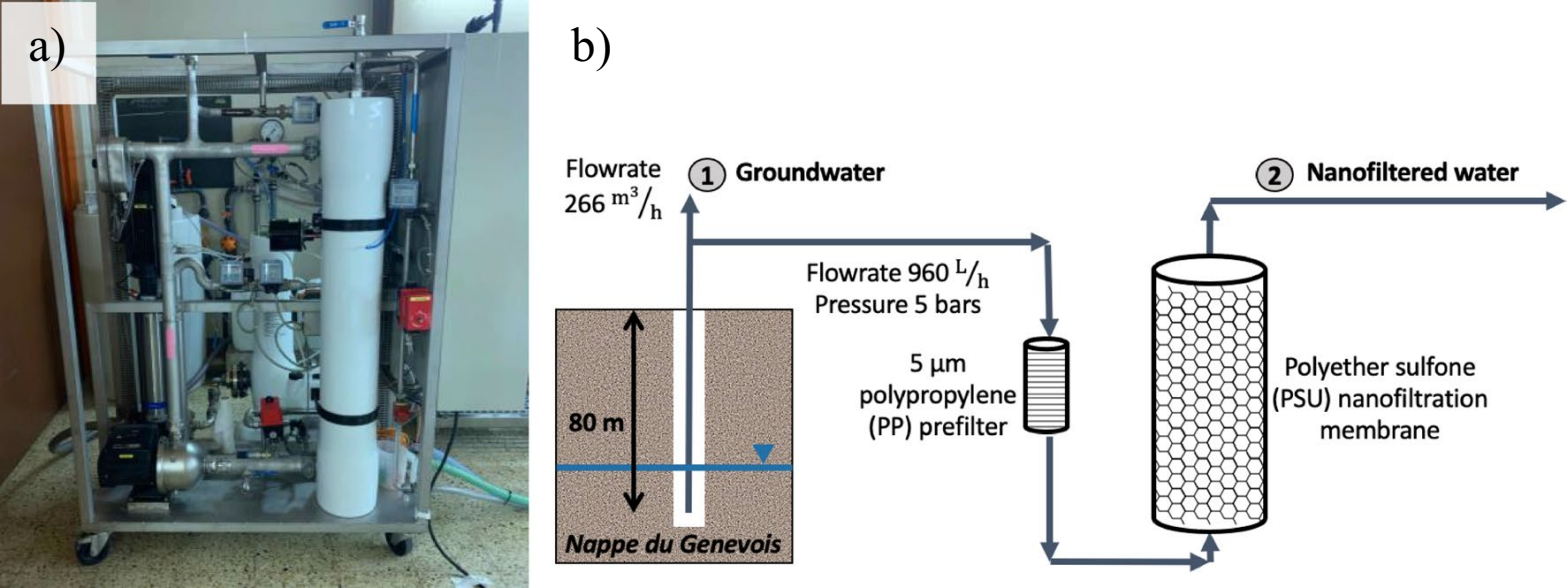
**a** Image of the nanofiltration system and **b** scheme of the drinking water pilot plant with marked sampling sites. (1) groundwater and (2) nanofiltered water

The pumping water plant of Soral was chosen to investigate the occurrence of MPs in groundwater and the output of a pilot nanofiltration system. Soral plant is located in an agricultural zone in Geneva, near France — Switzerland border and extract water from 80-m depth. Since abnormally high traces of perchlorate have been detected in Geneva’s groundwater in recent years, the station of Soral is out of operation. Therefore, the tests were carried out in a provisional pilot plant, which was installed mainly in the framework of several tests carried out to treat groundwater. Groundwater flowrate for the groundwater samples was fixed at 266 m^3^/h, in a water installation made of ethylene propylene diene monomer (EPDM) rubber hose and polyvinyl chloride (PVC) pipes (Fig. 1a and Fig. S1a). The nanofiltration pilot station was installed in Soral’s plant and had a flow of 960 L/h at a mean pressure of 5 bars. A PVC hose was used to connect the groundwater installations to the nanofiltration system. The connections of mobile nanofiltration system (Fig. 1b) were made of inox pipes. The nanofiltration treatment consists of a prefiltration membrane made of polypropylene (PP) fibres and a polyethersulfone membrane in a spiral wound configuration with a membrane active area of 40.9 m^2^. Finally, a PVC hose was used to connect the nanofiltration system to a water tank, before which samples were taken (Fig. S1b).

### Sampling method

Sampling campaigns took place between March and June 2022. Groundwater and nanofiltered water were sampled 3 times each. On average, 830 L groundwater and 1000 L nanofiltered water were passed under gravity through a 100-mm-diameter stainless-steel sieve (ISO 3310–1) with 20-µm-mesh size, as shown in Figure S1. Field blanks (*N* = 5) were sampled in parallel with the samples under investigation. A 50-mm diameter-stainless-steel sieve (ISO 3310–1) with 20-µm-mesh size was placed next to the sampling sieve to assess the environment contamination (field blanks). Sieves were covered with aluminium foil during sampling time. After sampling, the stainless-steel sieves were wrapped in the same aluminium foil, stored in boxes and transported to the laboratory. No sample preparation was here applied and the retained material in the steel sieves were transferred to aluminium oxide (Al_2_O_3_) filters (Anodisc™, 0.2 µm, Ø 47 mm, GE Healthcare Life Sciences Whatman™, Buckinghamshire, UK) on the same day of sampling. The number of treatment steps was minimal to reduce the risk of cross-contamination with MPs. All filters were stored immediately in clean glass Petri dishes.

### Chemical identification of microparticles

Fourier transform infrared (FTIR) spectroscopy measurements were performed using a Spotlight 200i FTIR microscopy system (PerkinElmer). FTIR spectrometer is equipped with a mercury cadmium telluride (MCT) detector with aperture range between 10 and 100 µm. A spectral range between 600 and 4000 cm^−1^ was acquired, but due to the absorption properties of the Al_2_O_3_ filter, only the spectral range from 1250 to 4000 cm^−1^ was analysed. Spectral resolution was set at 4 cm^−1^ and 8 scans per spectrum were applied.

A quarter of the filter surface was analysed following the model used in Negrete Velasco et al. (2023) due to the time-consuming analysis. All microparticles were carefully observed and tested by FTIR, excepted when identified as mineral microparticles or microorganisms. Mineral microparticles or detritus of microorganisms were identified both visually and chemically by infrared microscopy.

The obtained spectra were compared with the reference spectra of Perkin Elmer polymer library and a homemade library. Spectra with Hit Quality Index (HQI) above 0.6 were considered high enough to be further analysed before determining whether the microparticles or fibres were indeed MP particles or synthetic fibres, respectively. Therefore, the results of the match were not systematically and automatically accepted when the HQI was above 0.6.

### Characterization of microparticles

Microparticles were divided in four classes of sizes (20–50 µm, 50–100 µm, 100–500 µm and 500–5000 µm). The size of the particles was measured at the maximum Ferret diameter. Moreover, microparticle classification was based on the shape and the nature. A distinction was made between MPs (including fragments, films, pellets, beads and foams) and synthetic fibres. Indeed, fibres are visually different from fragments (elongated), can be natural, semi synthetic, or synthetic and therefore constitute a specific type of microparticles, which were separated from the rest of MPs.

### Statistical analysis

The results from the blanks, the groundwater and the nanofiltered water sampling campaigns were checked for significance. To that end, ANOVA (one way analysis of variance) and Tukey’s HSD (honestly significant difference) post hoc test with a significance level of 0.05 were performed in Origin Pro. The results are presented in Fig. S2.

### Quality assurance and quality control

Several recent guidelines (Brander et al. 2020; Cowger et al. 2020; Schymanski et al. 2021) were taken into account to guarantee quality assurance. To avoid cross-contamination, no gloves were used and ultrapure water (Milli Q water, Millipore, Schaffhausen, Switzerland, with *R* > 18 MΩ·cm) was systematically filtered prior to use with nitrate cellulose filters (0.45 µm, Ø 47 mm, Sartorius Stedim Biotech, Göttlingen, Germany) in a glass filtration unit (VWR, Leuven, Belgium). The only plastic material used in the laboratory was the polytetrafluoroethylene (PTFE) washing bottle (Thermo Fischer Scientific, New York, USA). All glassware and sieves were washed with soap, natural bristles brushes and rinsed with filtered ultrapure water. All materials were covered with aluminium foil to avoid a possible air contamination. Only cotton clothing was worn, even under the cotton lab coat. A lint roller was used to remove all fibres attached to the clothes and lab coat. Moreover, all work surfaces were wiped down with ethanol before starting work, and the floor was mopped at least 2 times per week.

To ensure that the methodology here used meet quality requirements, a recovery rate (positive control) test was conducted with PE MPs (*N* = 80, PE powder of 95-µm-average particle size, Goovean fibres, England) that were mixed with 1.0 L filtered ultrapure water. In addition, laboratory working environment conditions were assessed with three procedural blanks (1.0, 2.0 and 10.0 L filtered ultrapure water).

## Results

### Recovery rate and blanks

The recovery rate resulted in 105% (21 out of the 80 PE MPs added were detected in a quarter of the filter surface), which could be considered as a good balance between the time-consuming analysis and representativeness of the samples. Moreover, positive control and blanks (field and procedural controls) revealed a contamination with PTFE MPs (most likely from the washing bottle), which were excluded from the results. On the other hand, between 0 and 3 MPs were detected in the field blanks (in total 5 phenoxy resin, 1 polypropylene and 1 polystyrene-based copolymers) and the laboratory controls (in total 4 phenoxy resin) (Tables S1 and S2). The limit of detection (LOD) ([20 µm] = 6 MPs) was calculated as the mean value of MPs identified in the procedural blanks (*µ* = 1.3) plus three times the standard deviation (*σ* = 1.5).

Synthetic fibres were not detected in any of the blanks (field nor laboratory). However, between 20 and 31 non-synthetic fibres (avg. 25 ± 6 fibres) were observed in laboratory controls. Higher numbers were observed in field blanks, between 72 and 12 non-synthetic fibres (avg. 32 ± 23 fibres). Some fibres in the blanks were successfully identified as cotton or cellulose, thanks to our reference material library.

### Microplastics in ground water and nanofiltered water

#### Microplastic concentration and size distribution

**Fig. 2:**
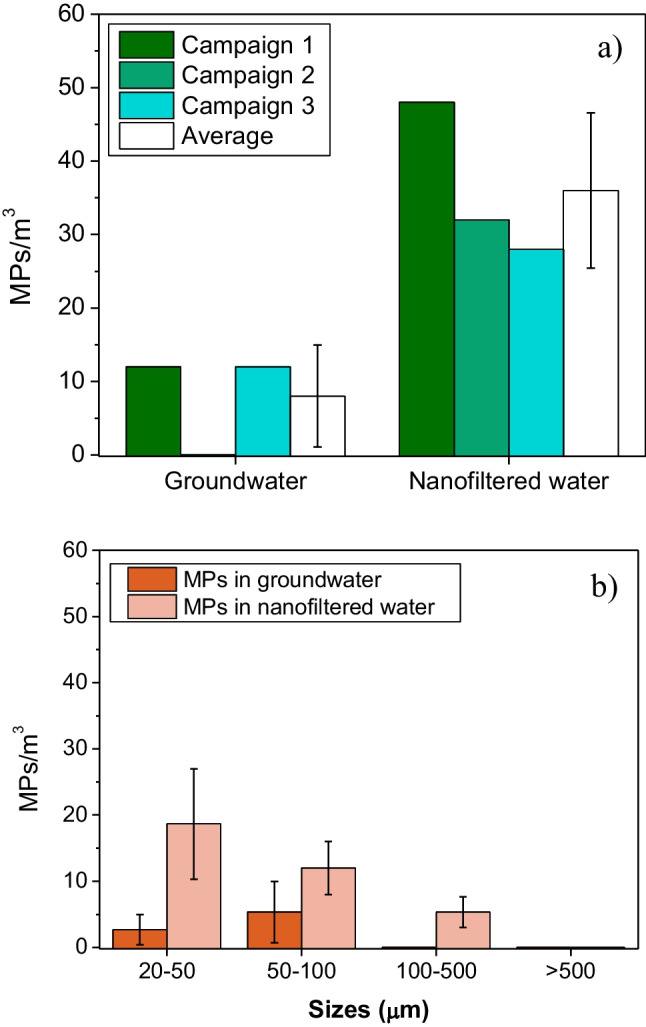
**a** Concentration of MPs and **b** size distribution of MPs in groundwater and nanofiltered water. Error bars represent standard deviations from the three sampling campaigns

MPs were found in groundwater and in nanofiltered water (Table 1). A total of 6 MPs were detected in a quarter of the filters surface in groundwater samples (833 L) and 27 MPs in nanofiltered water (3000 L) in the three sampling campaigns. The calculated average concentration of MPs in groundwater was equal to 8 ± 7 MPs/m^3^ (Fig. 2a), whereas average calculated MP concentration in nanofiltered water equal to 36 ± 11 MPs/m^3^, was found higher (Fig. 2).

**Table 1:**
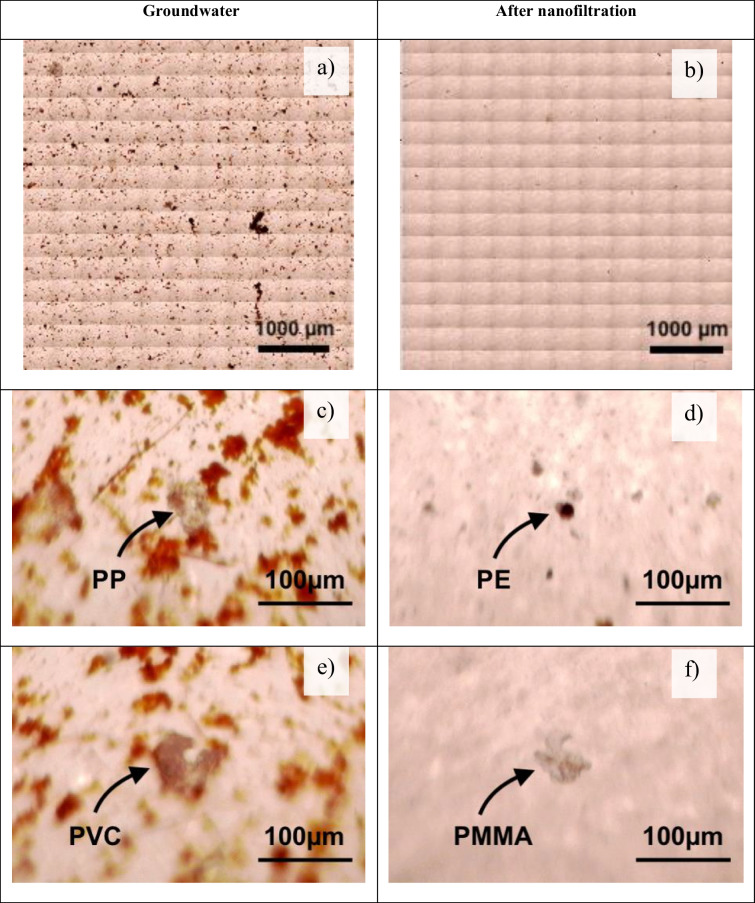
(a) Surface of the Al_2_O_3_ filter of the groundwater sample and (b) the nanofiltered water sample and (c–f) examples of MPs obtained with IR microscope found in groundwater (left column), nanofiltered water (right column)

All MPs had a maximum Ferret diameter < 500 µm. Size distribution of MPs was towards the small size class (20–50 µm), as shown in Fig. 2. In groundwater, 33% of MPs were detected in the smallest size class (20–50 µm) and 67% in the 50–100-µm-size class. In comparison, around 52% of MPs in nanofiltered water were observed in the 20–50-µm-size class. Moreover, 33% of the MPs observed in nanofiltered water were in the 50–100-µm-size class and 15% in the 100–500-µm-size class.

### Chemical composition of microplastics

**Fig. 3:**
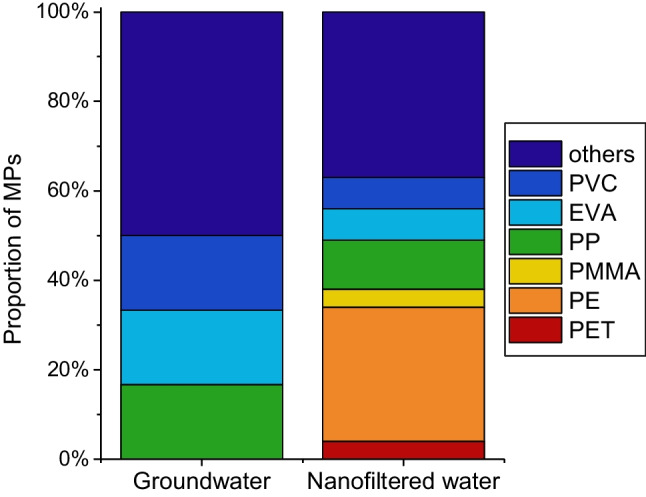
Chemical composition of MPs in groundwater and nanofiltered water. PE-polyethylene, PET-polyethylene terephthalate; PMMA, polymethyl methacrylate; PP, polypropylene; EVA, ethylene (vinyl acetate) copolymer; PVC, polyvinyl chloride and materials with more than one match are summarized as “others”

For all samples, different materials were identified by infrared spectroscopy, such as PP, PVC, ethylene (vinyl acetate) copolymer (EVA), other polymer materials (such as vinyl-based and polystyrene-based copolymers, such as acrylonitrile butadiene styrene and butadiene styrene) and phthalate plasticisers (Table S3). In addition, polyethylene (PE), polyethylene terephthalate (PET) and polymethyl methacrylate (PMMA) were identified in nanofiltered water (Fig. 3).

### Fibres in groundwater and nanofiltered water

#### Fibre concentrations and size distribution

**Fig. 4:**
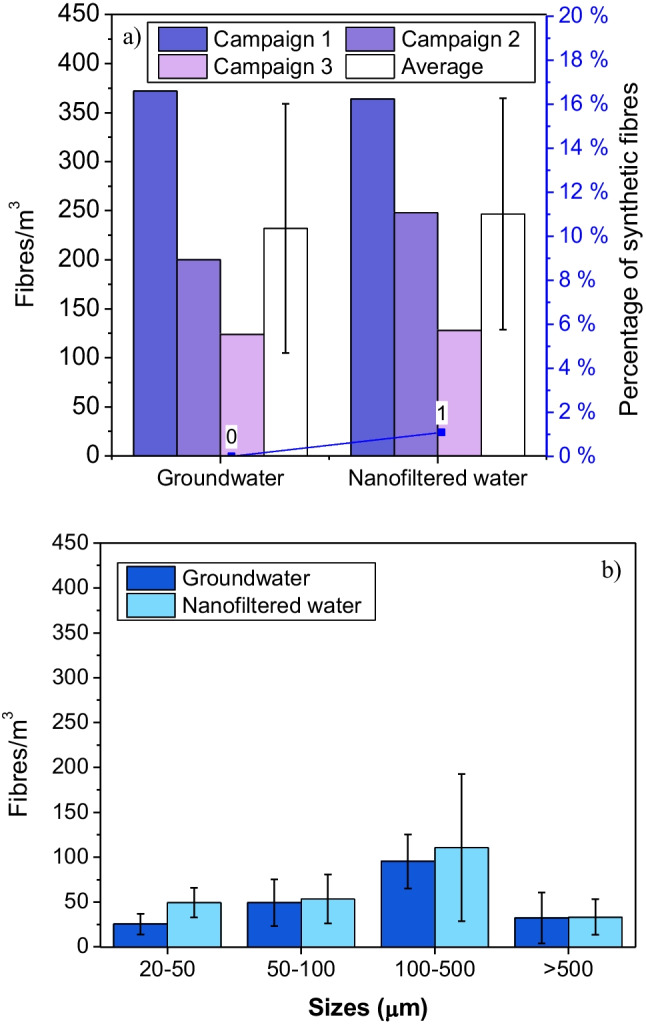
**a** Concentration of fibres and average percentage of synthetic fibres and **b** size distribution of fibres in groundwater and nanofiltered water. Error bars represent standard deviations from the three sampling campaigns

Fibres were observed in all samples and no clear difference of fibre concentrations was observed between groundwater (232 ± 127 fibres/m^3^) and nanofiltered water (247 ± 118 fibres/m^3^). Size distribution of fibres was different than for MPs. Near 45% of fibres belonged to the 100–500-µm-size class and around 15% of fibres were observed in the 500–5000-µm-size class (Fig. 4b). A small number of fibres (near 1%) were identified as PP synthetic fibres in nanofiltered water (Fig. 4).

## Discussion

### Strengths and limitations

Since low numbers of MPs were expected in groundwater and nanofiltered water, large water volumes were sampled to have representative samples (> 500 L) and therefore obtaining better results. The stainless-steel sieve (ISO 3310–1) of 100-mm diameter with 20-µm-mesh size allowed us to sample large volume samples of groundwater and nanofiltered water without clogging. In addition, the flow rate of the nanofiltration pilot station (960 L/h) passed successfully through this sieve size without overflowing. Sample fractioning with several sieves was considered unnecessary in this study, since most of MPs do not have regular forms and because it is of common and good practice to measure MPs size by microscopy (Schymanski et al. 2021; De Frond et al. 2022).

Low-sample contamination occurred during handling through atmosphere deposition and laboratory equipment. Phenoxy resin was the only polymer identified in the procedural blanks (and detected in some of the samples) even though no use was made of this material. However, airborne sample contamination during sampling was considered preferable than the use of filter housing units, which highly contaminate samples (Mintenig et al. 2019). Moreover, sample contamination occurred here with PTFE MPs due to the washing bottle. Nevertheless, being aware of the use of this material, all PTFE MPs were excluded from the results since this is not a very common plastic. Different volumes (1.0, 2.0 and 10.0 L) were used in procedural blanks to evaluate the contamination, but no significant difference was observed between procedural blanks. Field and procedural blank samples’ results were not presented in concentration and were rather considered as the number of MPs contributed to the samples.

Low numbers of PVC MPs were identified in groundwater and nanofiltered water. Similar concentrations of this plastic type were found in both kinds of samples, but no more than one PVC MP per sample was found. Sample contamination by PVC pipes could be considered a possibility since no PVC MPs was found in the blanks. Therefore, the results suggest that MPs detected in groundwater were rather from atmospheric deposition, the use of plastic pipes and hoses and sample handling than from the occurrence of MPs in groundwater.

Average number of MP detected in groundwater (2 ± 2, *N* = 3) was below LOD ([20 µm] = 6 MPs), while the number of MPs detected in nanofiltered water samples (9 ± 3, *N* = 3) were above LOD ([20 µm] = 6 MPs). Moreover, no significant difference was found between blanks (procedural and field) and groundwater (*p* > 0.05). However, nanofiltered water was significantly different from the rest (*p* < 0.05) (Fig. S2). The results suggest therefore a contamination in nanofiltered water with MPs due to the nanofiltration system.

High content of suspended particulate matter was observed in groundwater samples, which are naturally present in abstracted groundwaters. The number of suspended particulate matter (most likely iron or manganese oxides) was significantly reduced by nanofiltration (Table 1a and b). Indeed, the presence of this suspended particulate matter in groundwater hindered optical characterisation and increased the analysis time. However, no sample treatment (complexation/digestion) was applied with the aim of having similar methodologies in both sample types. Moreover, we intend to reduce the risk of sample contamination with MPs as much as possible by reducing the number of treatment steps. Despite the lack of standardization of the methodology, several guidelines were followed to ensure that the analysis was performed under quality assurance and quality control criteria. Our work was assessed with the total accumulated score (TAS) (Hermsen et al. 2018; Koelmans et al. 2019), which is used as an indicator of the reliability of the study. Maximum achievable score (TAS) is 18, and our score was equal to 14 because laboratory work was not performed under clean room or laminar flow cabinet; no blank correction was applied (because this practice is not recommended in unanimity in all guidelines); no replicates from positive controls were carried out and because a quarter of the filters was analysed. This score could be considered as relatively good, compared to other studies in the literature (Koelmans et al. 2019), proving the high quality assurance and quality controls in this study.

### Comparison with other studies

Currently there is no harmonization of MP extraction and analysis, so the comparison of results obtained here and in other studies is mainly based on certain elements, like water source, nanofiltration applied for drinking water, size range distribution and analysis technique (FTIR).

Mintenig et al. (2019) investigated ground water in Germany by sampling from 300 to 1000 L and found low concentrations of MPs (between 0 and 7 MPs/m^3^). Using FTIR imaging, they were capable to identify MPs down to 20 µm. However, all MPs were detected in a size ranged between 50 and 150 µm. Johnson et al. (2020) studied groundwater in a chalk and sandstone aquifer in Wales and found concentrations of MPs ranging from no detected (LOD [25 µm] = 139 MPs) to 11 MPs/m^3^. In contrast, Samandra et al. (2022) detected (LOD [20 µm] = 9 MPs) higher concentrations of MPs (from 16 to 97 MPs/L) in ground water in different areas in Australia. In this alluvial unconfined aquifer in Australia, the MP sizes ranged from 18 to 491 μm (avg. 89 ± 55 μm). Greater MP concentrations (from 10 to 34 MPs/L) were observed in a shallow unconfined aquifer in Sinaloa when analysing particles (LOD [63 µm] = 6.5 MPs) down to 63 µm by ATR (Alvarado-Zambrano et al. 2023). However, it is important to highlight that 1 L of ground water was sampled by Samandra et al. (2022) and Alvarado-Zambrano et al. (2023), whereas several hundreds of litres were sampled by Mintenig et al. (2019), Johnson et al. (2020) and this study, as summarized in Table 2. On the other hand, no blank subtraction of the samples was applied in this study as recommended in recent guidelines on MPs (Brander et al. 2020; Cowger et al. 2020; Schymanski et al. 2021), whereas Mintenig et al. (2019), Johnson et al. (2020), Samandra et al. (2022) and Alvarado-Zambrano et al. (2023) subtracted the blank value from the samples.

**Table 2:** Summary of scientific articles dealing with MP detection in groundwater

Reference	Type of aquifer	Volume sampled	Analytical technique	MP concentrations	MP sizes investigated (µm)	Limit of detection, LOD
Mintenig et al. (2019	No information	0.3–1 m^3^	FTIR	0–7 MPs/m^3^	50–150	No information
Johnson et al. (2020	Chalk and sandstone	1.5–4.3 m^3^	FTIR	11 MPs/m^3^	> 25	[25 µm] = 139 MPs
Samandra et al. (2022	Alluvial sediments (sand, gravel, silt, and clay)	1 L	Agilent novel laser direct infrared (LDIR)	16–97 MPs/L	18–491	[20 µm] = 9.0 MPs
Alvarado-Zambrano et al. (2023	Alluvial, fluvial, aeolian, lacustrine and sediments, and polymictic conglomerates (sand, silt, and clay)	1 L	ATR	10–34 MPs/L	63–1002	[63 µm] = 6.5 MPs
This study	Glacial and fluvioglacial sediments, (sand, gravel, and silts)	0.5–1 m^3^	FTIR	0–12 MPs/m^3^	20–500	[20 µm] = 6.0 MPs

Recently, Barbier et al. (2022) investigated MP contamination in three conventional drinking water treatment plants with surface water sources, including one nanofiltration system. MPs were detected (LOD [25 µm] = 8 MPs) in two out of six samples of nanofiltered water (less than 1 MP/m^3^ on average). The median size of MPs was 60 µm, and no MP contamination from the nanofiltration membrane (polypiperazine-amide and polyethersulfone) was found. Kara et al. (2022) reported a significant abatement of MPs (99%) in the landfill leachate treatment plant in Turkey. MP concentration after the nanofiltration was determined as 2 MPs/L. In contrast, Zhang et al. (2021) studied the efficiency of a leachate treatment process (membrane bioreactor, two-stage anoxic/oxic, ultrafiltration, nanofiltration and reverse osmosis) to remove MPs and found that MP concentration decrease from 1.2 (influent of the treatment) to 0.3 (after ultrafiltration). However, the advanced treatment techniques (nanofiltration and reverse osmosis) were not found to improve MP removal but increased the number of MPs due to the composition of the membranes, which released MPs. In comparison, we studied the impact of nanofiltration in the groundwater of Geneva, which contamination with MPs could be considered as limited. Our results indicate a minor contamination with MPs and synthetic fibres in nanofiltered water. The main component of the spiral wound membrane is polyethersulfone (which was not found); however, the cartridge is made of several layers (permeate spacer, membrane and feed spacer) and components (supporting materials, permeate collection tube and seals) of other plastic materials, which could release low number of MPs to nanofiltered water.

## Conclusion

The occurrence of MPs in an almost confined aquifer and in a nanofiltration pilot plant system was here assessed. Large volumes (500–1000 L) were sampled using a stainless-steel sieve of 20 µm. Size distribution and chemical composition of MPs were performed by infrared microscopy. MP concentrations in Geneva’s groundwater (8 ± 7 MPs/m^3^) were lower than nanofiltered water (36 ± 11 MPs/m^3^). However, low numbers of MPs were detected in groundwater (below the limit of detection), and it can be considered that the contamination with MPs in the groundwater of Geneva is limited. Based on these results, we can estimate that the inhabitants of Geneva (who are supplied with water from the Genevese aquifer) would be consuming at least 4 MPs (≥ 20 µm) per year from drinking tap water (considering an average daily consumption of 1.5 L of water). This intake via drinking water could be considered low compared to the 883 particles/capita/day consumed per adult (Mohamed Nor et al. 2021). Nevertheless, the presence of MPs down to 1 µm need to be further studied to better assess the exposure. Moreover, it is important to highlight that there is a disconnection between environmental research and toxicological tests. Indeed, most of the research on the toxicity of MPs to mammals has been conducted with polystyrene spheres, which represent a small fraction of the MPs observed in freshwaters and in drinking water (Coffin et al. 2022). Toxicity posed by particles depends on several physical properties (such as size, surface area and shape) and on the chemical composition of the particles (World Health Organization 2019). Therefore, further research with MPs of different sizes, shapes and chemical composition is needed to fill the knowledge gaps on the toxicological effects of MPs under real conditions.

Suspended particulate matter has been significantly removed from groundwater by nanofiltration treatment; however, the nanofiltration system and other plastic components of the pilot unit (pipes, valves and containers) are made of plastic materials and may slightly contaminate water with MPs. An important question is whether in a real groundwater installation we could expect to have higher MP contamination because of the larger contact surfaces (pipes, etc.) and whether higher speeds and pressures improve the release of MPs. Therefore, one precaution could be to replace certain elements of the nanofiltration system with no plastic materials to avoid mechanical stress on plastic materials and MP release. Apart from that, no clear results have been obtained regarding fibre contamination because of airborne contamination. However, MP concentration values here obtained are in the same order of magnitude as other studies.

### Supplementary Information

Below is the link to the electronic supplementary material.

Supplementary file1 (DOCX 3717 KB)

## Data Availability

Not applicable.
