# Supplementary material for: Impact of a nanofiltration system on microplastic contamination in Geneva groundwater (Switzerland)
Source: Environ Sci Pollut Res Int. 2024 Jan 23;31(9):13512–22. doi: 10.1007/s11356-024-31940-y (PMC10881595; doi:10.1007/s11356-024-31940-y)
Supplement: Supplementary file 1 — Supplementary file1 (DOCX 3717 KB) [file 11356_2024_31940_MOESM1_ESM.docx]

**Supplementary Material for:**

**Impact of a nanofiltration system on microplastics contamination in Geneva groundwater**

Angel Negrete Velasco ^a*^, Alicia Ellero^b^, Stéphan Ramseier Gentile ^b^, Stéphane Zimmermann ^b^, Pascal Ramaciotti ^b^ and Serge Stoll ^a.^

^a^ Department F.-A. Forel for environmental and aquatic sciences, University of Geneva, Faculty of Science, Group of Environmental Physical Chemistry, 66, boulevard Carl-Vogt, CH-1211 Geneva 4, Switzerland.

^b^ SIG, Industrial Boards of Geneva, Switzerland

* Corresponding author: Angel Negrete Velasco

angel.negretevelasco@unige.ch

Université de Genève

Département F.-A. Forel des sciences de l’environnement et de l’eau.

Groupe de Physico-Chimie de L'Environnement.

Université de Genève– Boulevard Carl-Vogt 66

CH-1211 Genève 4, Switzerland.

Keywords: microplastics, fibres, groundwater, nanofiltration, infrared microscopy, suspended particulate matter.


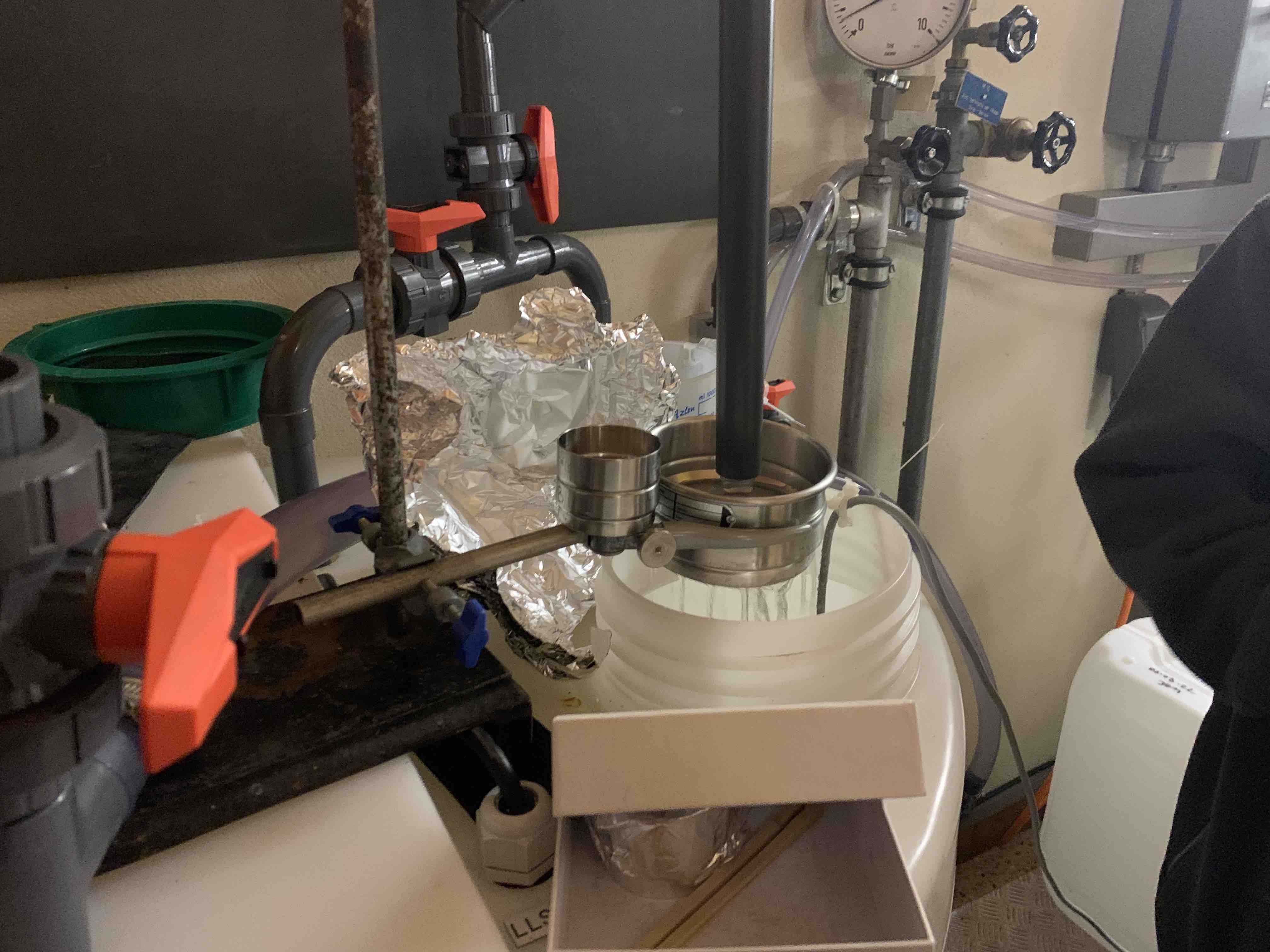

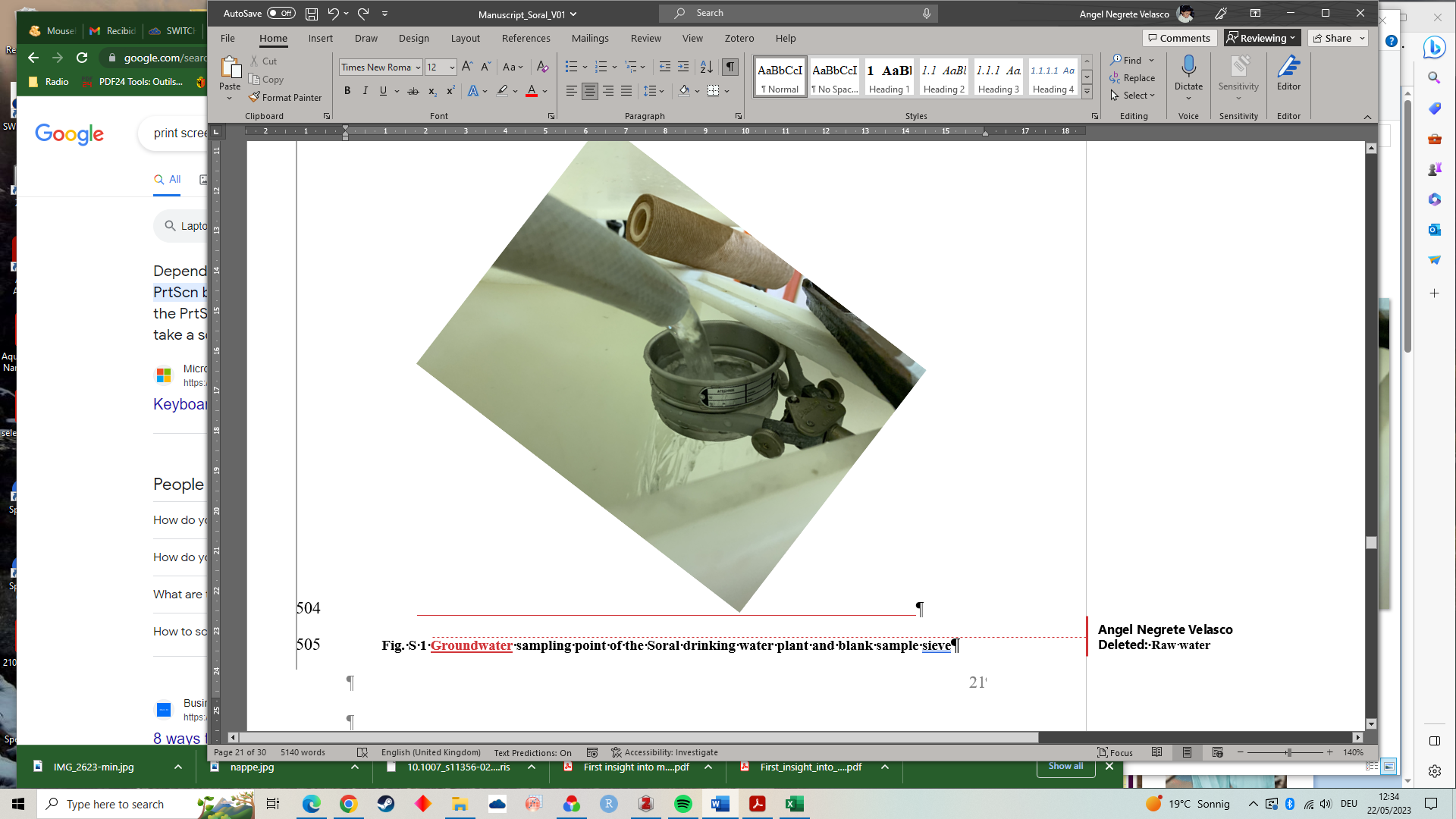


b)

a)

Fig. S 1 a) groundwater sampling point of the Soral pumping groundwater plant and blank sample sieve and b) sampling sieve of the nanofiltered water.


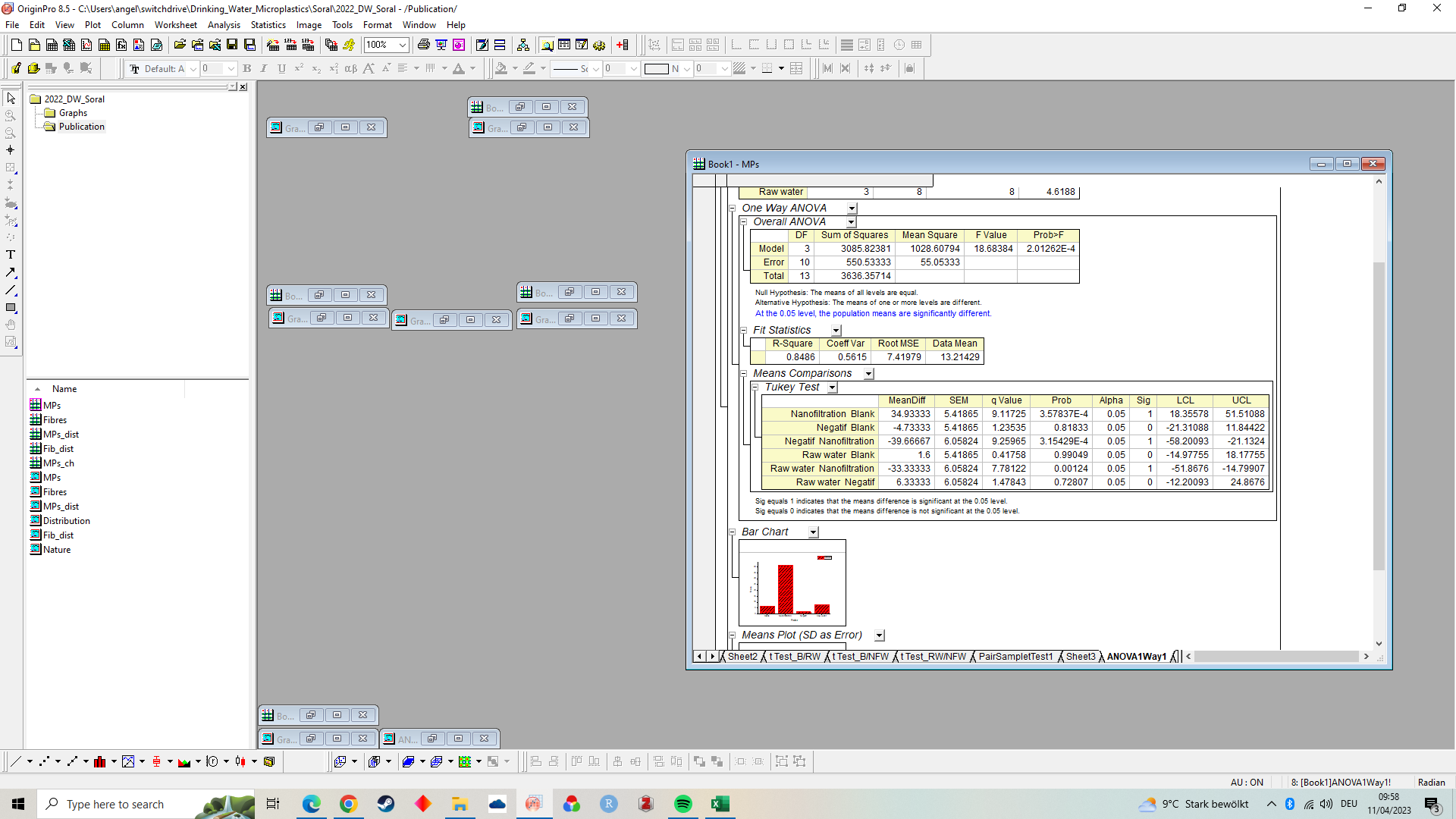


Fig. S 2 one way ANOVA test and Tukey’s test results from 3 groundwater sampling campaigns, 3 nanofiltered water sampling campaigns, 3 procedural blanks and 5 field blanks.

| Sample µm | Field blank 1 | Field blank 2 | Field blank 3 | Field blank 4 | Field blank 5 | Average | Standard Deviation |
| --- | --- | --- | --- | --- | --- | --- | --- |
| 20-50 | 0 | 3 | 2 | 1 | 0 | **1.2** | **1.3** |
| 50-100 | 0 | 0 | 0 | 1 | 0 | **0.2** | **0.45** |
| 100-500 | 0 | 0 | 0 | 0 | 0 | **0.0** | **0.0** |
| >500 | 0 | 0 | 0 | 0 | 0 | **0.0** | **0.0** |
| **Total MPs** | **0** | **3** | **2** | **2** | **0** | **1.4** | **1.3** |

Table S. 1 number of MPs detected in blank field samples (MPs counts per filter).

| Sample µm | Procedural blank 1 (1L) | Procedural blank 2  (2L) | Procedural blank 3  (10L) | Average | Standard Deviation |
| --- | --- | --- | --- | --- | --- |
| 20-50 | 1 | 0 | 3 | **1.3** | **1.5** |
| 50-100 | 0 | 0 | 0 | **0.0** | **0.0** |
| 100-500 | 0 | 0 | 0 | **0.0** | **0.0** |
| >500 | 0 | 0 | 0 | **0.0** | **0.0** |
| **Total MPs** | **1** | **0** | **3** | **1.3** | **1.5** |

Table S. 2 number of MPs detected in laboratory procedural blank samples (MPs counts per filter).

| Polymer | HQI | Spectra |
| --- | --- | --- |
| PE | 0.77 | 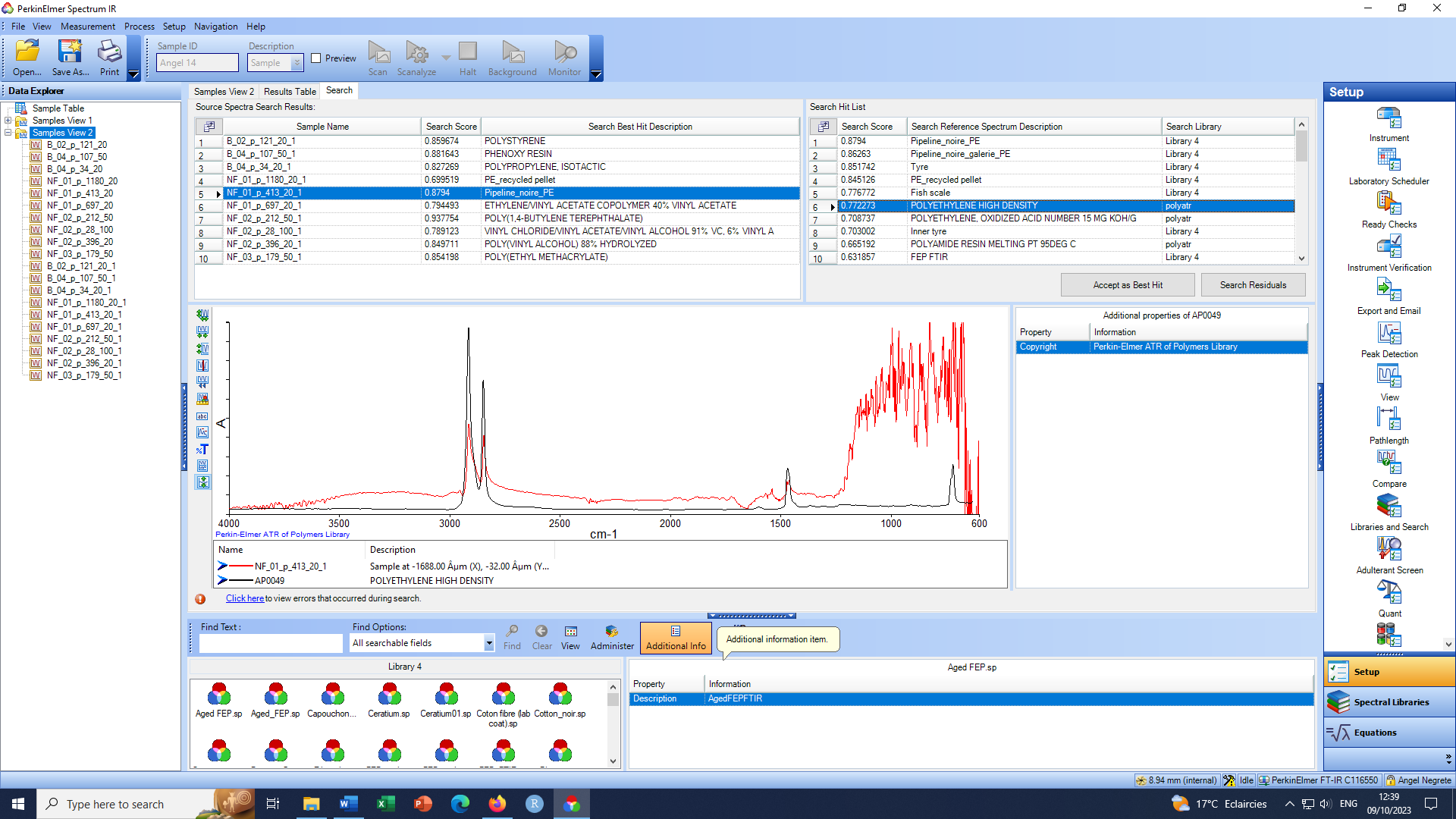 |
| PET | 0.68 | 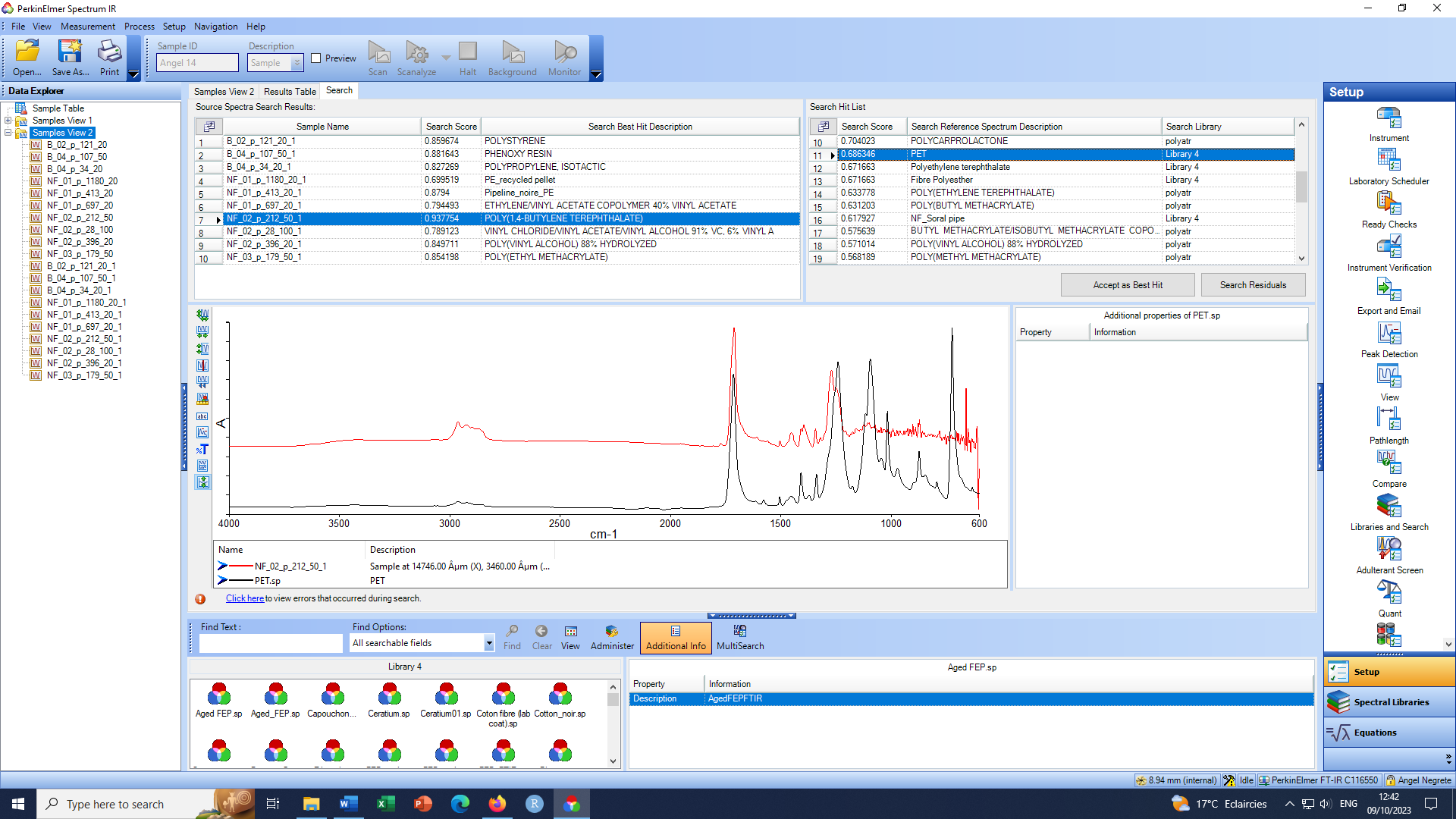 |
| PMMA | 0.85 | 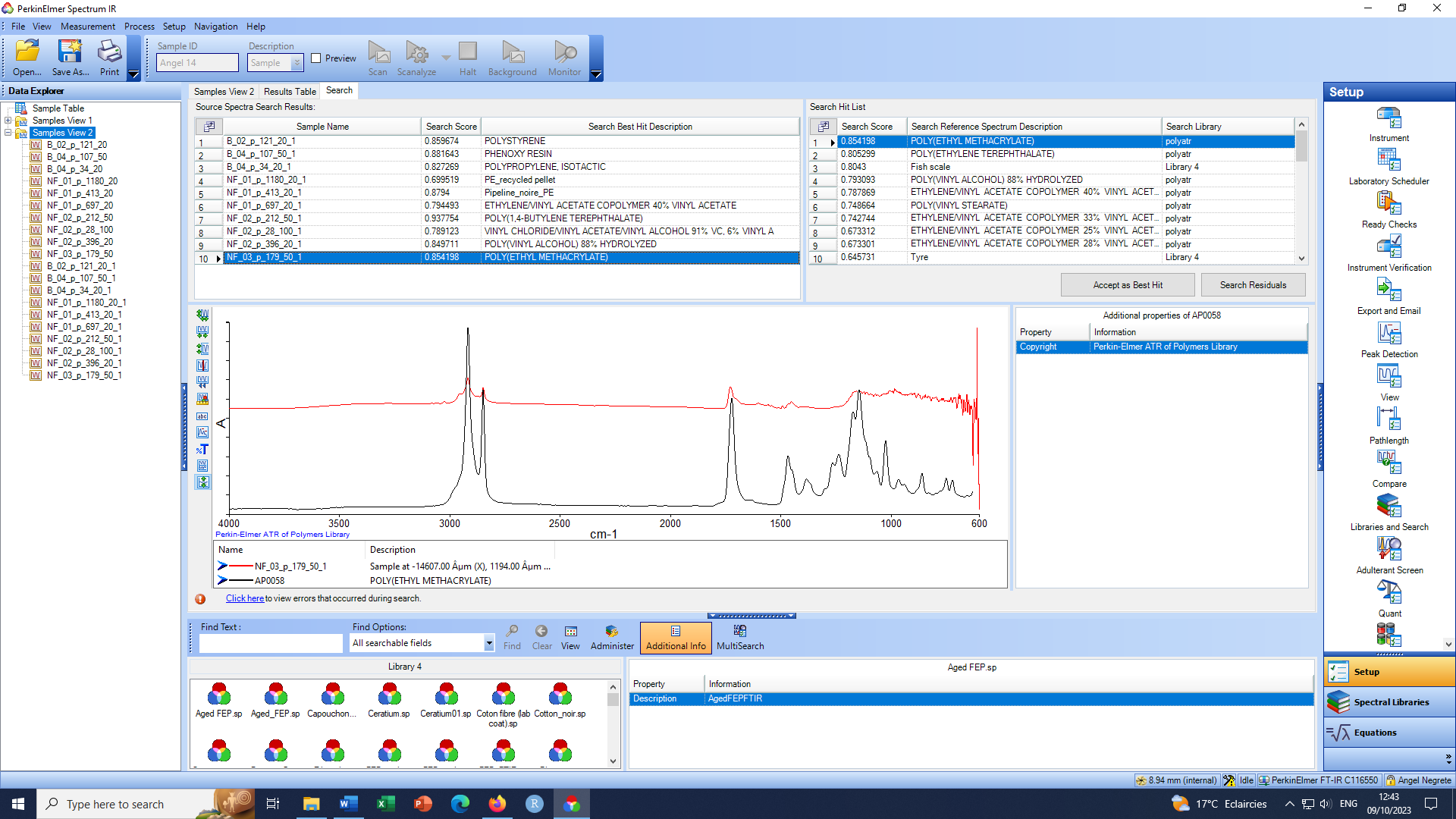 |
| PP | 0.83 | 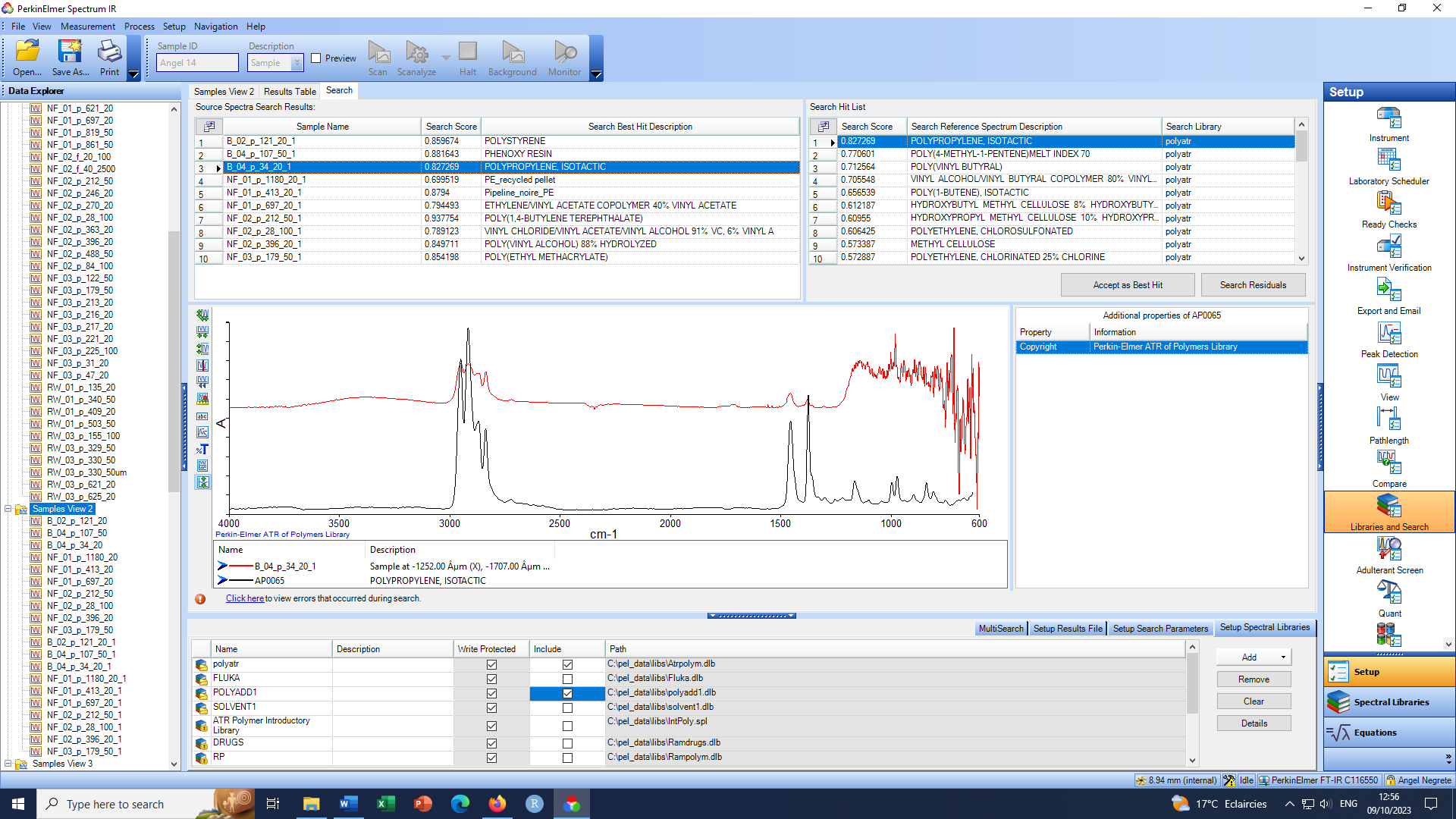 |
| PS based copolymer ( | 0.82 | 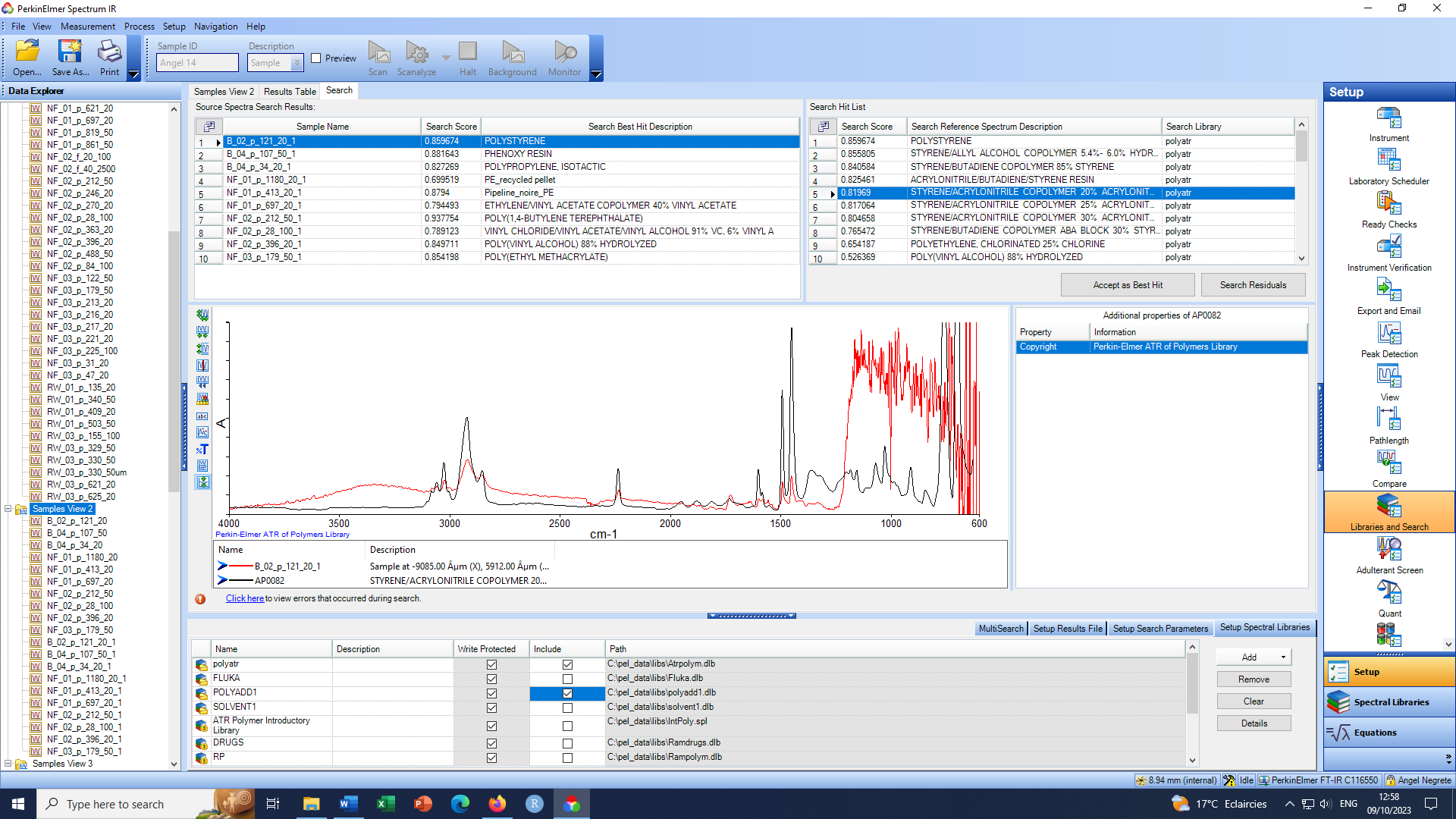 |
| EVA | 0.79 | 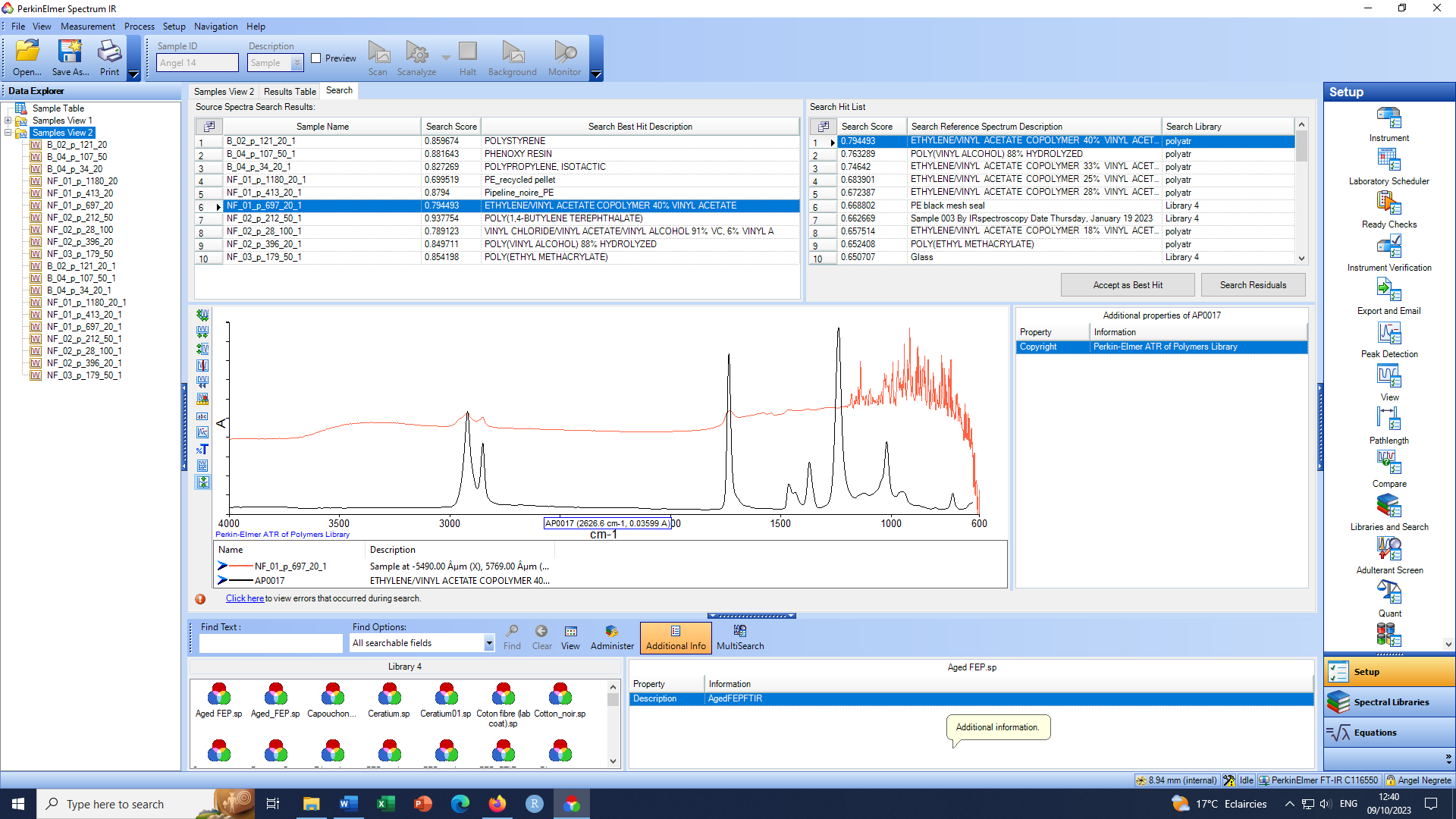 |
| PVC | 0.82 | 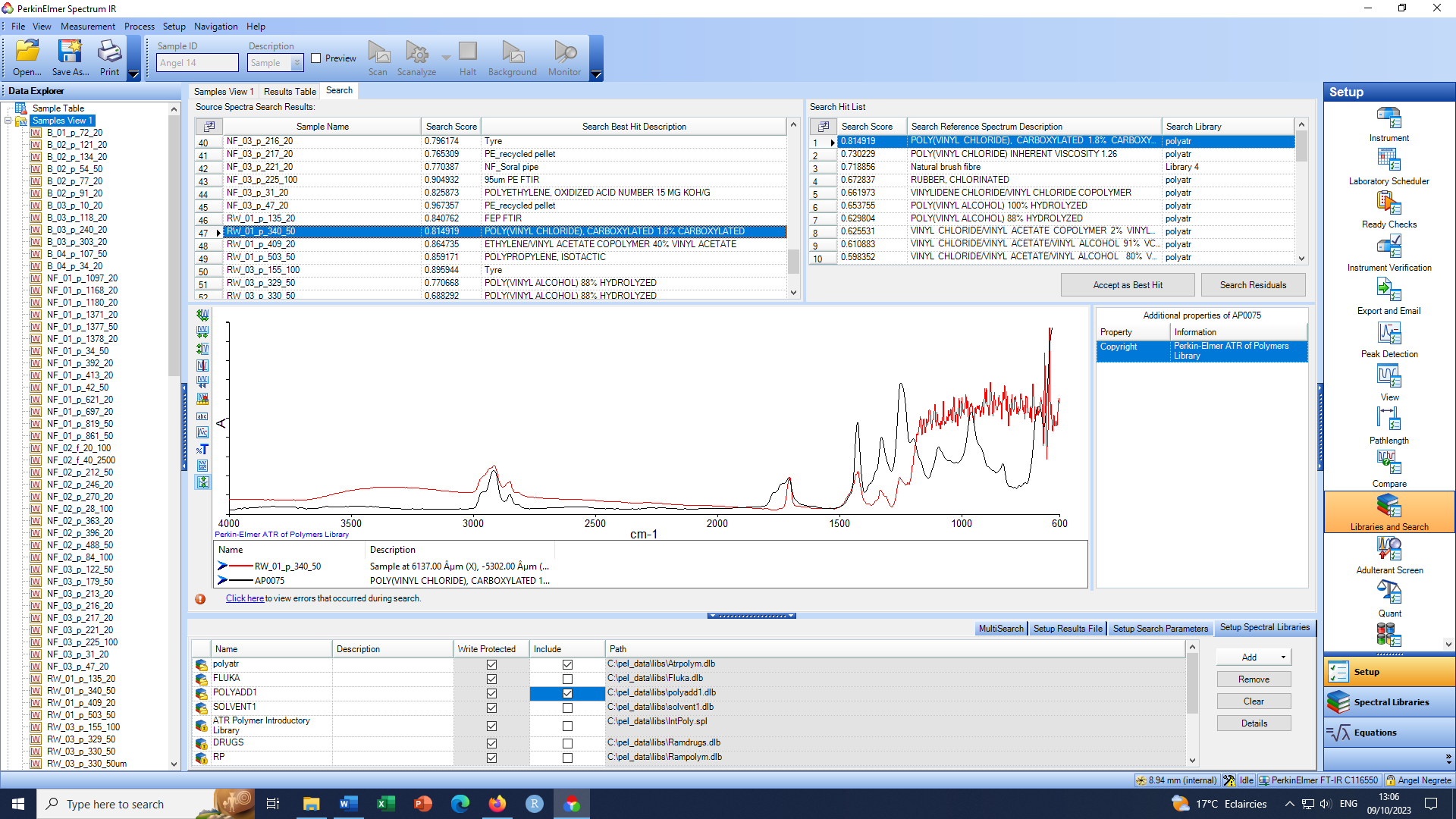 |
| Phenox | 0.84 | 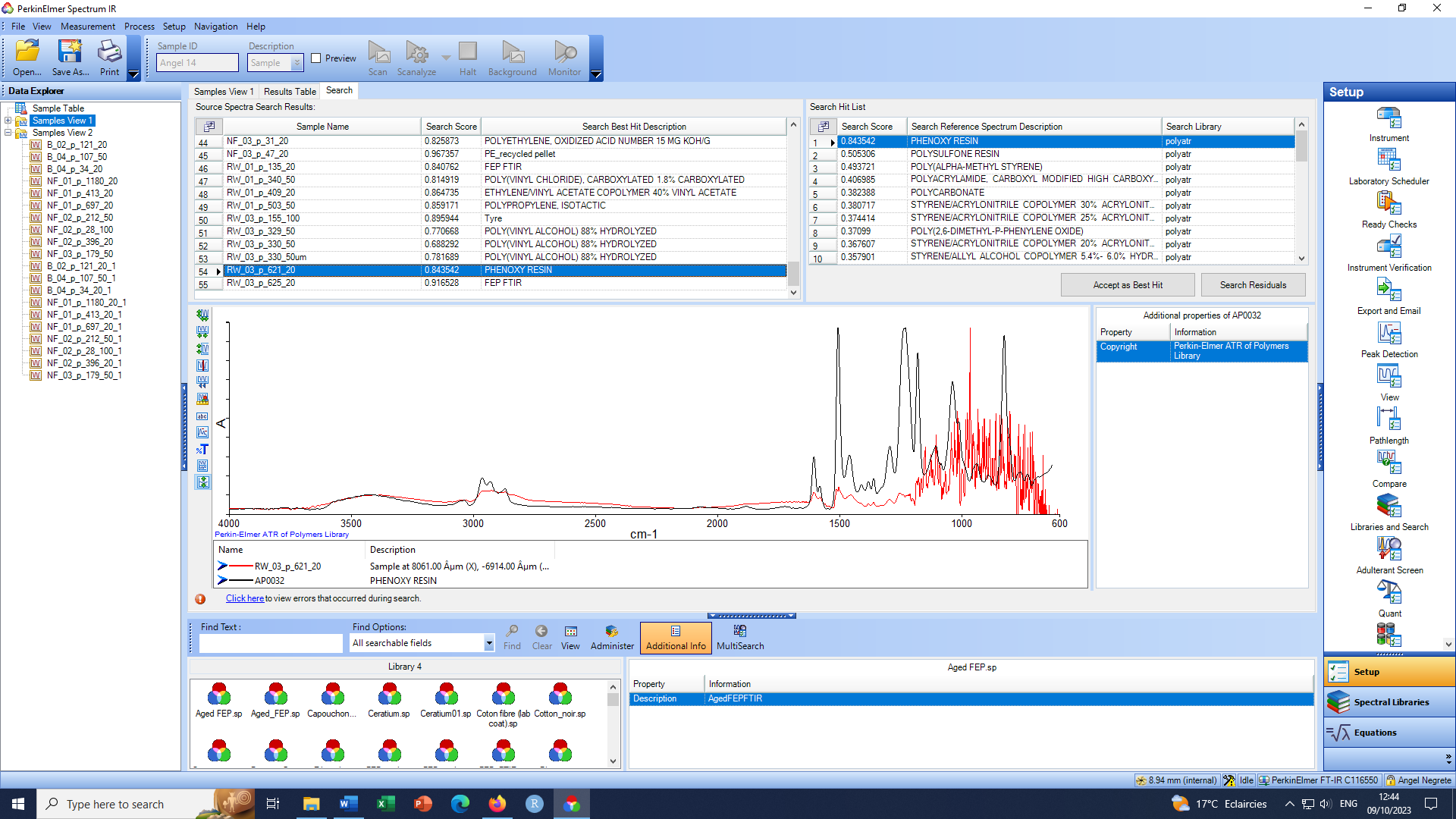 |
| Poly(Diallyl isophthalate) | 0.81 | 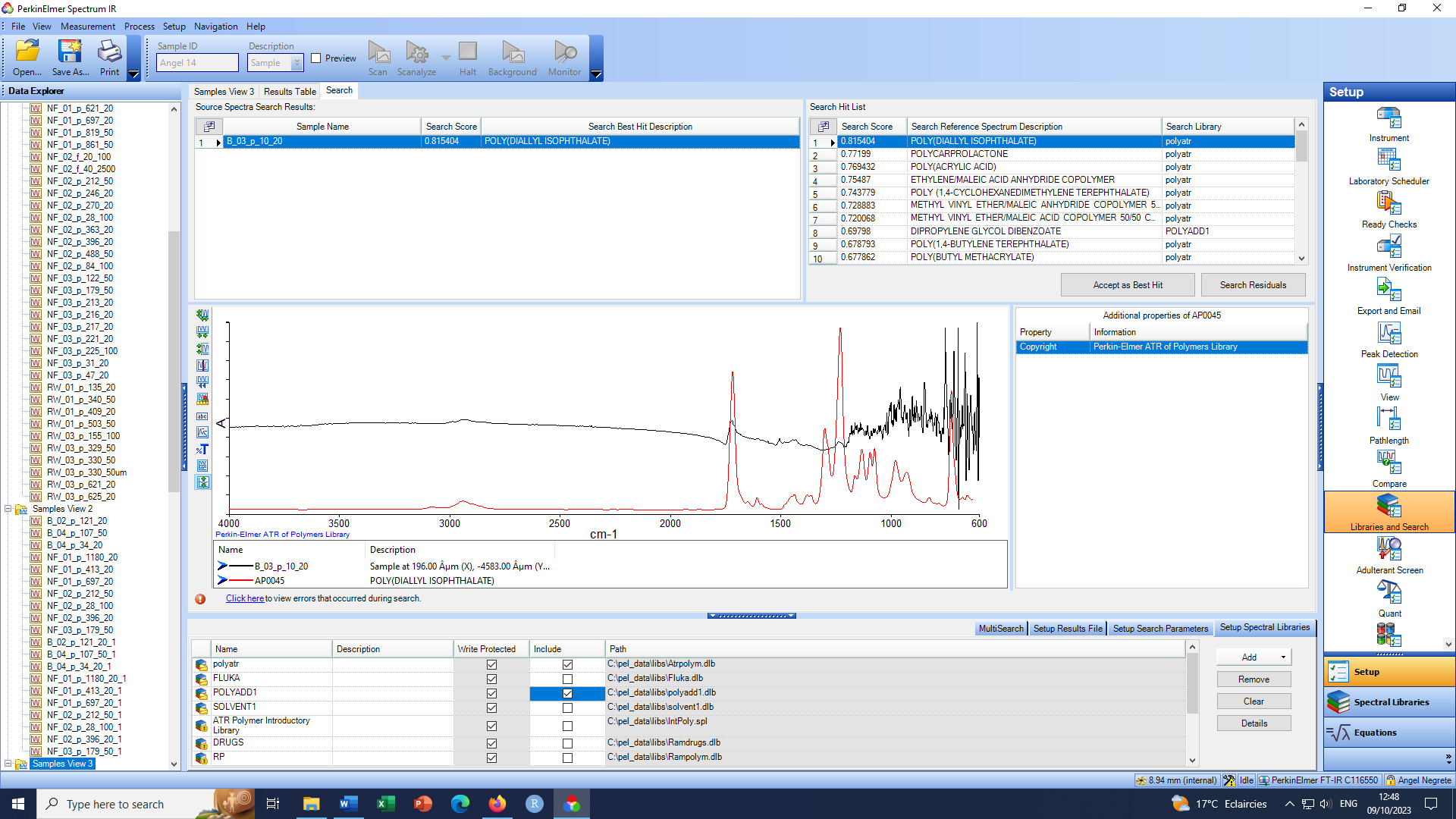 |

Table S. 3 Polymers identified by Fourier transform infrared spectroscopy. Red lines are the measured spectra and black lines are the reference spectra.
